# The *sf32* Unique Gene of *Spodoptera frugiperda* Multiple Nucleopolyhedrovirus (SfMNPV) Is a Non-Essential Gene That Could Be Involved in Nucleocapsid Organization in Occlusion-Derived Virions

**DOI:** 10.1371/journal.pone.0077683

**Published:** 2013-10-30

**Authors:** Inés Beperet, Gloria Barrera, Oihane Simón, Trevor Williams, Miguel López-Ferber, Laila Gasmi, Salvador Herrero, Primitivo Caballero

**Affiliations:** 1 Instituto de Agrobiotecnología, CSIC-UPNA-Gobierno de Navarra, Mutilva Baja, Navarra, Spain; 2 Corporación Colombiana de Investigación Agropecuaria (CORPOICA), Bogotá, Colombia; 3 Instituto de Ecología AC, Xalapa, Veracruz, Mexico; 4 Ecole des Mines d’Alès, Alès, France; 5 Department of Genetics, Univesitat de València, Burjassot, Valencia, Spain; 6 Departamento de Producción Agraria, Universidad Pública de Navarra, Pamplona, Navarra, Spain; CSIR-Central Drug Research Institute, India

## Abstract

A recombinant virus lacking the *sf32* gene (Sf32null), unique to the *Spodoptera frugiperda* multiple nucleopolyhedrovirus (SfMNPV), was generated by homologous recombination from a bacmid comprising the complete viral genome (Sfbac). Transcriptional analysis revealed that *sf32* is an early gene. Occlusion bodies (OBs) of Sf32null contained 62% more genomic DNA than viruses containing the *sf32* gene, Sfbac and Sf32null-repair, although Sf32null DNA was three-fold less infective when injected *in vivo*. Sf32null OBs were 18% larger in diameter and contained 17% more nucleocapsids within ODVs than those of Sfbac. No significant differences were detected in OB pathogenicity (50% lethal concentration), speed-of-kill or budded virus production *in vivo*. In contrast, the production of OBs/larva was reduced by 39% in insects infected by Sf32null compared to those infected by Sfbac. The SF32 predicted protein sequence showed homology (25% identity, 44% similarity) to two adhesion proteins from *Streptococcus pyogenes* and a single N-mirystoylation site was predicted. We conclude that SF32 is a non-essential protein that could be involved in nucleocapsid organization during ODV assembly and occlusion, resulting in increased numbers of nucleocapsids within ODVs.

## Introduction

The family *Baculoviridae* comprises a group of viruses characterized by a large double-stranded, circular, supercoiled DNA molecule of 80–180 Kb, enveloped in a rod-shaped nucleocapsid [1]. These viruses are pathogenic to arthropods, particularly Lepidoptera, and have a number of characteristics that favor their development as bioinsecticides, such as restricted host range and high pathogenicity and virulence [2], [3]. Baculoviruses are present in the environment, in soil and on foliage, as occlusion bodies (OBs), which are formed by a protein matrix that occludes the occlusion derived virions (ODV). Two phases can be distinguished in their cycle of infection. Primary infection occurs when the larva ingests OBs that degrade in the alkaline midgut and release ODVs that infect epithelial cells. Virus replication and transcription begins in midgut cells and new nucleocapsids are formed in the cell nucleus. Some of these nucleocapsids bud out of the cells acquiring an envelope as they do so, and form budded virions (BV), that disperse within the infected insect to initiate secondary infection. The nucleocapsids that remain in the nucleus of infected cells acquire an external envelope, either singly or in groups, to form the ODVs that are occluded in the polyhedrin matrix to form the occlusion bodies. Upon death the insect disintegrates and liquefies and OBs are released into the environment for transmission to susceptible larvae [1]. As such BVs are specialized for cell-to-cell systemic infection, whereas ODVs are required for insect-to-insect transmission.

The fall armyworm, *Spodoptera frugiperda*, is an important pest of maize, rice and sorghum in North and South America. This insect can be infected by *S. frugiperda* multiple nucleopolyhedrovirus (SfMNPV) and natural epizootics of virus disease can spread through high density populations of the pest [4]. Several SfMNPV isolates have been characterized [5]–[7], but development of the virus as a biological insecticide has been limited, mainly due to high production costs and moderate levels of pest control observed following application of viral occlusion bodies (OBs) to infested crops [8], [9]. The identification of the genetic factors that determine particular insecticidal properties of the virus may facilitate the selection of particular genotypes with desirable traits for use in bioinsecticidal products, or the development of recombinant viruses, with improved characteristics compared to the wild type [10]. The deletion of certain genes [11] or the insertion of insect-specific toxin genes [12] has been shown to increase the speed of kill of these viruses.

Studies on the genes involved in the insecticidal characteristics of SfMNPV have been facilitated by genome sequencing of three different isolates of this virus [13]–[15]. A total of 62 open reading frames (ORFs) present in these genomes have homologues in all lepidopteran-specific baculoviruses (genus *Alphabaculovirus*) [16]. Most of these genes encode structural proteins or are involved in DNA replication or transcription [17]. The majority of SfMNPV genes show high sequence homology to genes of *Spodoptera exigua* multiple nucleopolyhedrovirus (SeMNPV). However, there are twelve open reading frames (ORFs) in the SfMNPV genome that have been identified as unique genes that do not have homologs in other baculoviruses sequenced to date [13], [14]. These genes may play a role in the unique characteristics or host specificity of SfMNPV [18], [19].

The *sf32* gene of SfMNPV is a unique gene located in a hypervariable region of the genome within which mutations and deletions influence viral phenotype [13], [20]. In the present study, we examined the role of this gene in the insecticidal properties of this virus. A PCR and a bacmid-based recombination system were used to delete *sf32* from the genome and a selection of phenotypic characteristics of the recombinant product was studied. We found that the *sf32* gene is not essential for the SfMNPV infection cycle, as infectious viral progeny were obtained following replication of the sf32 deletion bacmid. However, deletion of the gene caused a reduction in OB production per larva, whereas the size of OBs and the number of viral genomes (nucleocapsids) within ODVs increased. We suggest that SF32 may be involved in nucleocapsid organization during ODV assembly and occlusion.

## Material and Methods

### Insects, Cells and Viruses

*Spodoptera frugiperda* larvae were obtained from a colony that was started using pupae from a laboratory population maintained in Honduras and refreshed periodically with pupae from southern Mexico. The colony was maintained at 25±1°C, 75% relative humidity (RH) and 16 h light: 8 h dark photoperiod on a wheatgerm-based semisynthetic diet [21]. *Spodoptera frugiperda* Sf9 cells were maintained in TC100 medium containing 10% fetal calf serum at 28°C [22]. A wild-type isolate of SfMNPV was collected in Nicaragua and characterized genotypically by Simón et al. [23]. The B genotype (SfMNPV-B) includes the largest genome of the virus. This genotype was selected for the SfMNPV bacmid construction (Sfbac). The complete genotype was cloned into a pBACe3.6 vector modified by replacing the pUC19 element with pBluescriptKS I containing *Asc*I restriction sites, which cuts the SfMNPV-B genome once [24].

### Construction of Sf32null and Sf32null-repair Viruses

The Sf32null bacmid was constructed by deleting *sf32* from Sfbac by homologous recombination using Red/ET recombination (Gene Bridges GmbH). A kanamycin resistance gene was amplified twice using the Tn5-neo PCR template and primers that added 50 nucleotide (nt) terminal sequences corresponding to either 3′ or 5′ untranslated regions of *sf32*. First, a PCR fragment was amplified using Sf32del.1 and Sf32del.2 primers (Table 1) and the Tn5-neo template. Then, in a second PCR, 25 nt terminal sequences were added using Sf32del.3 and Sf32del.4 primers (Table 1) and the PCR product of the first amplification. The bacteria containing Sfbac were made electrocompetent and transformed with the Red/ET plasmid pSC101-BAD-gbaA (Gene Bridges GmbH). The PCR product containing the terminal sequences of the *sf32* gene was used to transform the electrocompetent cells containing Sfbac and pSC101-BAD-gbaA. These cells were also made electrocompetent and induced with arabinose (0.1–0.2% w/v) to express the recombination protein (gbaA). Recombinants were selected as resistant colonies on medium containing chloramphenicol and kanamycin. To confirm deletion of *sf32*, restriction *Pst*I profiles of the bacmid DNA were examined and PCR amplifications with Sf32del.3 and Sf32del.4 primers were sequenced.

**Table 1:** Primers used in this study.

Primers	Sequences	Amplification purpose
Sf32del.1	5′-ATCATTATATTGCTTTGTATTTTATGGACAGCAAGCGAACCGGAA-3′	*Sf32* deletion from SfMNPV bacmid; forward primer with 24 nt homologous to Tn-5neo sequence (underlined) and 21 nt homologous to 3′ untranslated *sf32* region (nt 30,932–30,955 in SfMNPV-B genome).
Sf32del.2	5′-AATTTTTTTTATATTTGGGCATAGTCTCAGAAGAACTCGTCAAGA-3′	*Sf32* deletion from SfMNPV bacmid; reverse primer with 19 nt homologous to Tn-5neo sequence (underlined) and 26 nt homologous to 5′ untranslated *sf32* region (nt 31,363–31,338 in SfMNPV-B genome).
Sf32del.3	5′-GGAAAAGTTGTGTAAATAAAACAACATCATTATAATGCTTTGTAT-3′	*Sf32* deletion from SfMNPV bacmid; forward primer with 20 nt homologous to Sf32del.1 primer (underlined) and 25 nt homologous to 3′ untranslated region (nt 30,907–30,931 in SfMNPV-B genome).
Sf32del.4	5′-TTATTAGAAAATTAAGAAAAGTTCAATTTTTTTTATATTTGGGCA-3′	*Sf32* deletion from SfMNPV bacmid; reverse primer with 21 nt homologous to Sf32del.1 primer (underlined) and 24 nt homologous to 5′ untranslated region (nt 31,383–31,364 in SfMNPV-B genome).
Sf32rep.1	5′-CGCTATTGTTAGCGACACGA-3′	*Sf32* insertion into Sf32null bacmid; forward primer that amplifies 809 pb upstream the *sf32* gene (nt 30148–30167 in the SfMNPV-B genome).
Sf32rep.2	5′-GGTGCGATACGATCAATGTG-3′	*Sf32* insertion into Sf32null bacmid; reverse primer that amplifies 1348 pb downstream the *sf32* gene (nt 32804–32823 in the SfMNPV-B genome).
Sf32.1	5′-AAGTGGATGCCGATAAAACG-3′	*Sf32* transcription analysis (RT-PCR); forward primer that amplifies 280 bp upstream of the *sf32* stop codon (nt 31,243–31,224 in SfMNPV-B genome).
Sf32.2	5′-CCAATTGGTATGAATGCCAC-3′	*Sf32* transcription analysis (RT-PCR); reverse primer that amplifies in the *sf32* stop codon (nt 30,963–30,982 in SfMNPV-B genome).
Sfpolh.1	5′-CCCGACACCATGAAGCTGGT-3′	*Polh* transcription analysis (RT-PCR); forward primer that amplifies 500 bp upstream of the *polh* stop codon (nt 241–260 in SfMNPV-B genome).
Sfpolh.2	5′-TTAGTACGCGGGTCCGTTGTA-3′	*Polh* transcription analysis (RT-PCR); reverse primer that amplifies in the *polh* stop codon (nt 741–721 in SfMNPV-B genome).
egt.1	5′-TACGACCTGTTGCACCATAA-3′	*egt* transcription analysis (RT-PCR); forward primer that amplifies 479 bp upstream of the *egt* stop codon (nt 25154–25173 in SfMNPV-B genome).
egt.2	5′-TTACACAAAATTAAGTCTCA -3′	*egt* transcription analysis (RT-PCR); reverse primer that amplifies in the *egt* stop codon (nt 25633–25614 in SfMNPV-B genome).
qSf.1	5′-TGTGGTATATTTATGCACAGA-3′	BVs production (qPCR); forward primer that amplifies in the *sf68* (nt 63,179–63,199 in SfMNPV-B genome).
qSf.2	5′-ATTCAATGCTATCGTTTGAGC-3′	BVs production (qPCR); reverse primer that amplifies in the *sf68* (nt 63,279–63,259 in SfMNPV-B genome).

For the construction of the repair virus, the *sf32* coding region was amplified by PCR using primers amplifying outside the coding region, Sf32rep.1 and Sf32rep.2 (Table 1), and the Sfbac DNA as template. Fourth-instar *S. frugiperda* larvae were injected with 10 µl from a mixture containing 50 µl of Sf32null bacmid DNA (100 ng/ µl), 50 µl of the PCR product that covered the *sf32* region (500 ng/ µl) and 50 µl of Lipofectin reagent (Invitrogen). Inoculated larvae were transferred to diet and reared individually at 25°C. Virus-induced mortality was recorded daily. The OBs were purified from cadavers and virus DNA extracted as described below. A PCR was performed with Sf32rep.1 and Sf32rep.2 primers to determine whether recombination had replaced the kanamycin cassette with the *sf32* gene in the Sf32null bacmid. DNA was transfected into DH5α electrocompetent cells. In order to select colonies containing the *sf32* gene, bacmid DNAs were purified by alkaline lysis and restriction *Pst*I profiles and PCR amplifications with Sf32del.3 and Sf32del.4 were examined. PCR amplification products generated using Sf32del.3 and Sf32del.4 primers of the selected bacmid, were sequenced to confirm the correct insertion of the gene.

### Temporal Expression of *sf32*

Total RNA was isolated from Sfbac-infected larvae at 0, 2, 4, 6, 8, 10, 12, 24, 48, 72, 96, 120 and 144 hours post infection (hpi) with TRIzol reagent (Invitrogen) according to the manufacturer’s protocol. The extracted total RNA was treated with RNase-free DNase (Promega) to remove genomic DNA. First strand cDNA synthesis was performed using the Improm-II™ reverse transcriptase (Promega) and the internal oligonucleotide Sf32.1 (Table 1). The resulting cDNA mixtures were amplified using the *sf32*-specific primers Sf32.1 and Sf32.2 (Table 1). Amplifications of the very late and highly transcribed *polyhedrin* gene (*polh)* with Sfpolh.1 and Sfpolh.2 primers and the early *egt* gene with egt.1 and egt.2 primers (Table 1) were performed as a control. PCR products were subjected to electrophoresis in 1% agarose gel.

### Sfbac and Sf32null DNA Infectivity and Production of the OBs

Sfbac, Sf32null and Sf32null-repair bacmid DNAs were purified by alkaline lysis and caesium chloride gradient centrifugation [22]. To determine DNA infectivity and produce Sfbac, Sf32null and Sf32null-repair OBs, *S. frugiperda* fourth instars were injected with a DNA suspension including bacmid DNAs and Lipofectin reagent (Invitrogene) in a 2∶1 proportion [24]–[25]. A 100 µL volume of each bacmid DNA containing 100 ng/ µL was mixed with 50 µL of Lipofectin. After 10 minutes, 10 µL of this suspension was injected into individual larvae (666 ng/larva). Inoculated larvae were transferred to diet, reared individually at 25°C and virus mortality was recorded daily until death or pupation. Groups of 24 larvae were injected with DNA from each virus and the experiment was performed three times.

OBs obtained from dead larvae were filtered through cheesecloth, washed twice with 0.1% (w/v) sodium dodecyl sulphate (SDS) and twice with double-distilled water, and resuspended in double-distilled water. The resulting OB suspensions were counted in a Neubauer chamber and were stored at 4°C. To confirm the authenticity of the recombinant OBs, DNA was extracted from OBs as described in the following section and verified by restriction endonuclease and PCR analyses.

### DNA and ODV Content Within OBs

Genomic DNA was extracted from samples of 1×10^6^ OBs of Sfbac, Sf32null and Sf32null-repair bacmids. ODVs were released from OBs by mixing OB suspensions with 100 µL of 0.5 M Na_2_CO_3_ and 50 µL of 10% (w/v) SDS in a final volume of 500 µL and incubating at 60°C during 10 minutes. Undissolved OBs were removed by low speed centrifugation (3,800×*g*, 5 minutes). The supernatant fraction containing virions was treated with 25 µL of proteinase K (20 mg/mL) and incubated at 50°C for one hour. Viral DNA was extracted twice with 500 µL of phenol and once with chloroform and isolated by alcohol precipitation. The resulting pellet was resuspended in 50 µL of 0.1×TE buffer by incubation at 60°C during 10 minutes. DNA samples were diluted 1∶100 and quantified using qPCR based on SYBR green fluorescence in an ABI PRISM 7900HT Sequence Detection System (Applied Biosystems). The reaction mixture (20 µL) contained 10 µL SYBR Premix Ex Taq (2x), 0.4 µL of ROX Reference Dye (50x), 0.2 µL of each SfMNPV primer (10 pmol/ µL) (Table 1) and 1 µL of DNA template. qPCR was performed under the following conditions: 95°C for 30 s, followed by 45 amplification cycles of 95°C for 5 s and 60°C for 30 s and finally a dissociation stage of 95°C for 15 s, 60°C for 15 s and 95°C for 15 s. Data acquisition and analysis were handled by Sequence Detector Version 2.2.2. software (Applied Biosystems). Known dilutions of SfMNPV CsCl-purified DNA (10^−5^–1 ng/ µl) were used as internal standards for each qPCR reaction. Melting-curve analysis was performed to confirm specific replicon formation in qPCR.

Mean ODV content within OBs was determined by end point dilution in Sf9 cells [25]. ODVs were released from 5×10^8^ OBs in a volume of 500 µL by incubation with Na_2_CO_3_ 0.1 M at 28°C for 30 minutes. This suspension was filtered through a 0.45 µm filter and serially diluted 1∶5 in TC100 medium. Sf9 cells were infected with 10 µL of each ODV dilution in 96-well plates. Twenty-four wells containing 1×10^4^ cells/well were inoculated with each dilution in triplicate. Dishes were incubated at 28°C for 7 days and then examined for signs of virus infection. Results were analyzed by the Spearman-Kärber method [26] to estimate 50% tissue culture infectious dose (TCID_50_). TCID_50_ values were subsequently converted to infectious units per 5 10^8^ OBs and compared by t-test in SPSS 15.0 (SPSS Inc, Chicago, IL).

### Biological Activity of OBs

The 50% lethal concentration (LC_50_), mean time to death (MTD) and OB production were determined in second instars by *per os* inoculation following the droplet feeding technique [27]. To estimate LC_50_ values, larvae were starved overnight and allowed to drink viral suspensions in 10% (w/v) sucrose solution containing 0.001% (w/v) blue food dye and one of the following concentrations of OBs of each virus: 1.9×10^3^, 9.6×10^3^, 4.8×10^4^, 2.4×10^5^ and 1.2×10^6^ OB/ml. This range of concentrations was previously estimated to kill between 5 and 95% of the insects. Larvae that drank OB suspension in a 10 min period were reared individually on diet at 25°C and virus mortality was recorded daily until death or pupation. Bioassays were performed on four occasions using groups of 24 larvae per virus concentration and 24 control larvae that consumed sucrose and food dye solution without OBs. Virus induced mortality data were subjected to probit analysis with the POLO statistical program [28].

In order to determine speed-of-kill, groups of 24 recently-molted second instars were starved overnight and allowed to drink an OB concentration estimated to result in 90% mortality, namely, 2.19×10^5 ^OB/mL for Sfbac and 2.24×10^5 ^OB/mL for Sf32null. Larvae that drank OB suspensions within 10 min were reared individually on diet at 25°C and mortality was recorded at 8 hour intervals until larvae had died or pupated. The experiment was performed on four occasions. Time-mortality results were subjected to Weibull analysis using the generalized linear interactive modelling (GLIM) program [29] and to Kaplan-Meier survival analysis using SPSS 15.0.

OB production was determined in larvae that died in the MTD assays. Each corpse was homogenized in 100 µL distilled water and OBs were counted in a Neubauer hemocytometer. OB counts from each larva were performed in duplicate using three of the four replicates from the MTD experiment. Results were subjected to t-test in SPSS 15.0.

### Budded Virus Production *In Vivo*

Budded virus (BV) production was studied in newly-molted fourth instars that had drunk a suspension of 10^8 ^OB/ml, estimated to result in ∼95% mortality for both viruses. The number of viral genome copies present in larval hemolymph was estimated by qPCR. Hemolymph samples taken from groups of 20 larvae at 0, 12, 24, 48, 72, 96, 120 hpi were centrifuged at 2,000×*g* for 10 minutes at 4°C to pellet cells. DNA extraction was performed on the supernatant using the MasterPure Complete DNA Purification kit (Epicentre Biotechnologies) and DNA concentrations were measured by qPCR as described for DNA content quantification.

### Electron Microscopy

Scanning electron microscopy (SEM) was used to determine OB diameter. OBs in suspension were fixed overnight by mixing with an equal volume of fixative (4% formaldehyde and 1% glutaraldehyde in 0.1 M phosphate buffer, pH 7.4) and then washed twice with 0.1 M phosphate buffer. Samples were then partially dehydrated with ethanol 70%, dried, placed on aluminum mounts using carbon tags, sputter-coated with gold-palladium and photographed at magnifications of 6,000× and 25,000× using a scanning electron microscope (Philips SEM 550). Images were analyzed with the ImageJ software (National Institutes of Health) and Feret’s diameter (the longest distance between two parallel tangents) was taken as a measure of OB diameter. A total of 500 OBs were analyzed for the Sfbac virus and 345 were analyzed for the Sf32null virus. OB measurements were normalized by square-root transformation and compared by t-test using SPSS 15.0.

The number of ODVs occluded within OBs and the number of nucleocapsids per ODV were determined by examination of OB sections by transmission electron microscopy (TEM). OBs in suspension were fixed for 2 h at 4°C with 1.5% glutaraldehyde. Samples were then concentrated in 0.4% agar, washed with phosphate buffer (0.2 M, pH 7.3), post fixed with 2% osmium tetroxide for 2 h, followed by 1 h treatment with 2% uranyl acetate. Samples were then embedded in epoxy resin, sectioned, stained with lead acetate and observed under TEM at 100 KV (JEOL JEM 1010). Different fields of each sample were photographed at a magnification of ×40,000. Images were analysed using ImageJ software for each sample, the number of ODVs was counted in 30 OBs. Similarly, the number of nucleocapsids was counted in 300 ODVs. The mean numbers of ODVs and nucleocapsids for each sample were compared by t-test. Feret’s diameter was also measured in approximately 100 OBs of each virus (89 for Sfbac and 100 for Sf32null) and compared by t-test using SPSS 15.0.

### Gene and Protein Sequence Analysis

To determine the nature of the putative SF32 protein, nucleotide and amino acid sequences homologs were searched in the updated Genbank and EMBL databases using BLAST [30]. Protein properties were studied using the Peptide Property Calculator (Center for Biotechnology, Northwestern University). PSIPRED and JPRED3 tools were used to predict protein secondary structure [31] and signal sequences were screened using SIGNALP v3.0 [32]. Cellular location was predicted by TargetP 1.1 server [33]. The presence of transmembrane domains was detected using TMHMM v2.0 and MEMSAT3 [34]. Finally, post-translational modifications were predicted using PROSITE [35].

## Results

### Generation of Sf32null and Sf32null-repair Viruses

The selected Sf32null bacmid was expected to contain a deletion of the *sf32* gene, located between the nucleotides 30,955 and 31,338 in the SfMNPV-B genome [14]. Replacement of the *sf32* gene by the kanamycin cassette in Sf32null was confirmed by restriction endonuclease analysis and PCR with specific primers targeted at the predicted recombinant junction regions. The genomic arrangement of the recombinant virus was verified by sequencing. The same method was performed to confirm the correct insertion of *sf32* gene in the Sf32null-repair bacmid.

### *sf32* is an Early Transcribed Gene

**Figure 1:**
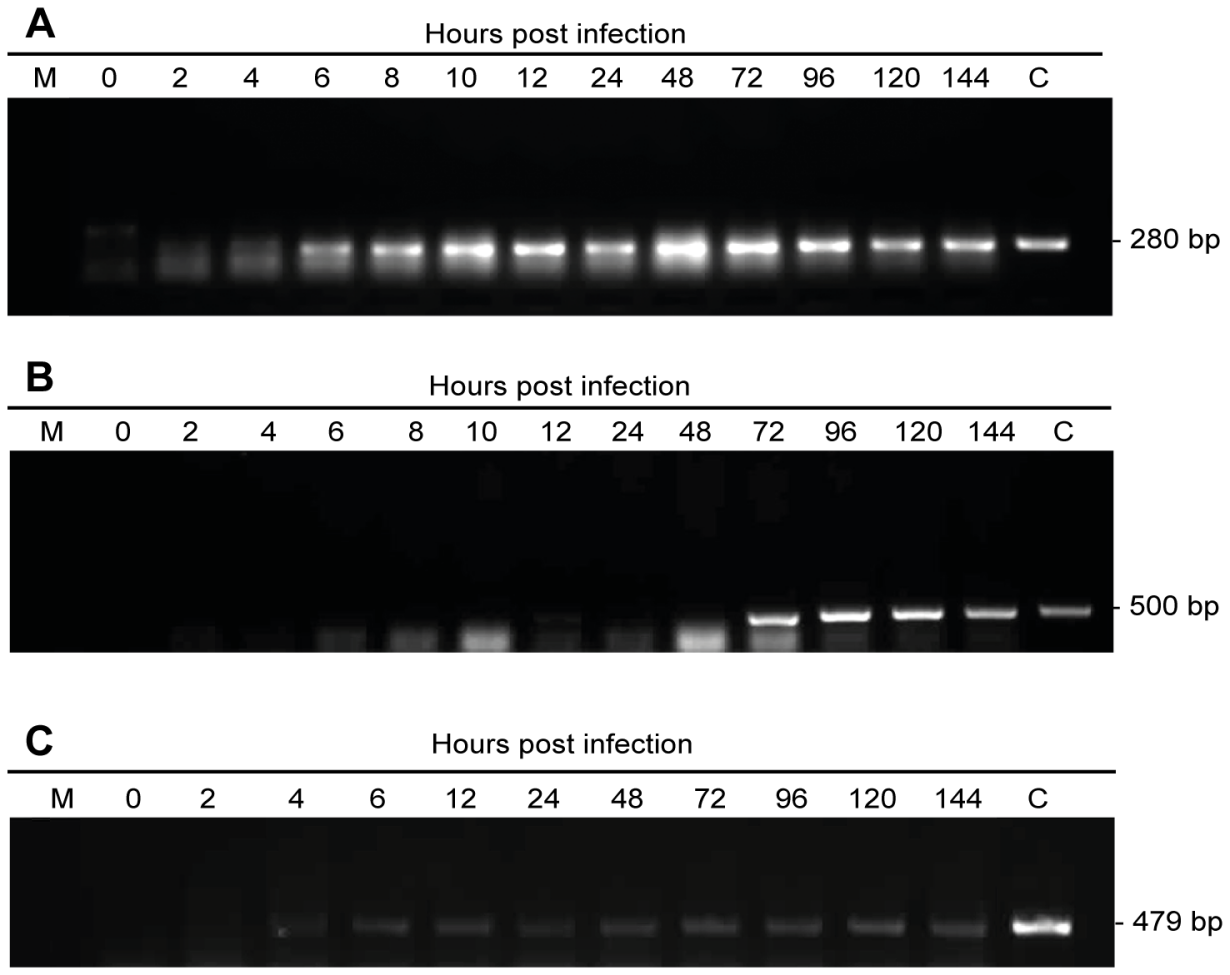
Temporal expression of a) *sf32*, b) polyhedrin (*polh*) and c) *egt* of SfMNPV. RT-PCR analysis of *sf32, polh* and *egt* was performed on total RNA extracted from infected larvae at indicated times post infection (hpi). Transcript amplifications were performed using Sf32.1 and Sf32.2 primers for *sf32*, Sfpolh.1 and Sfpolh.2 primers for *polh* and egt.1 and egt.2 primers for *egt*. RNA was previously treated with DNase and the same amount of RNA was used for *sf32*, *polh* and *egt* amplifications. M indicates RNA from mock-infected larvae (negative control) and C is a positive amplification control of DNA.

Temporal regulation of the *sf32* transcript was examined by RT-PCR using total RNA isolated from *S. frugiperda* larvae orally infected with Sfbac OBs. Control amplifications performed to ensure the absence of contaminant DNA in the samples were consistently negative. Equal volumes of the treated RNA were used for the *sf32*, polh and egt transcript amplifications. Single RT-PCR products of the expected sizes were obtained following amplification of *sf32*, *polh* and *egt*. The *sf32* amplification product was detected at a very low level at 2 hpi, increased at 4 hpi and remained at a steady-state level up to 144 hpi. In contrast, an amplification product from the early transcribed egt gene was detected from 4 hpi to 144 hpi. The late transcribed gene *polh* amplification product was detected at a very low level at 24 hpi, increased at 48 hpi and remained at a steady-state level up to 144 hpi (Figure 1). A diffuse band was observed below the expected amplification product due to excess primer.

### Reduced Infectivity of Sf32null DNA

**Figure 2:**
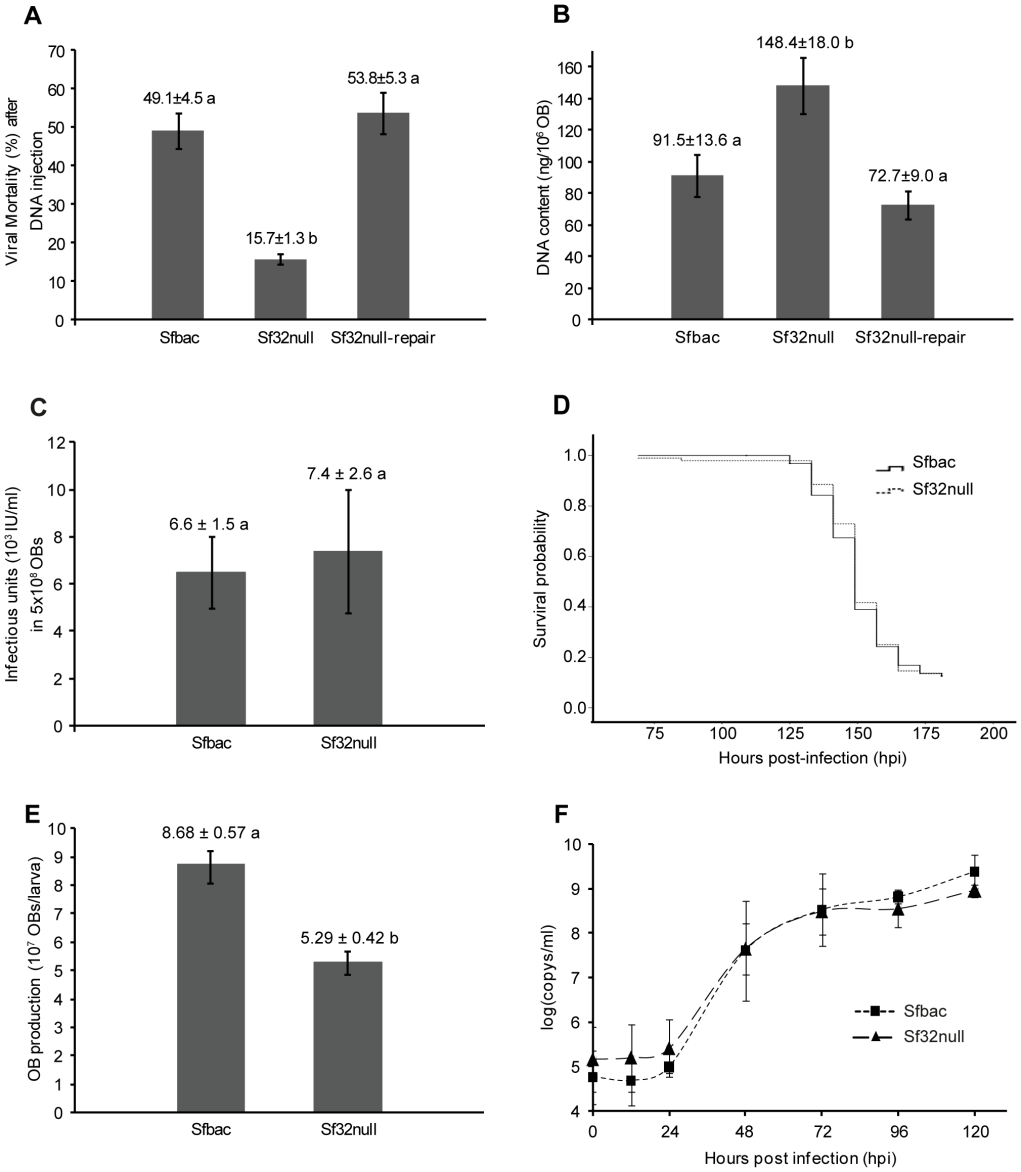
Effects of *sf32* deletion in the biological activity of the virus. a) Mean virus-induced mortalities following DNA injection. Values above the columns indicate means and those labeled with different letters are significantly different (p<0.05). Error bars indicate the standard error of the mean. Mortality was recorded until larvae had either died or pupated. b) Mean amounts of DNA extracted from samples of 10^6^ OBs of Sfbac, Sf32null and Sf32null-repair viruses. Values above columns indicate means and those labeled with different letters differed significantly (t-test, p<0.05). Error bars indicate the standard error of the mean. c) ODV content in 5×10^8^ OBs of Sfbac and Sf32null. Sf9 cells were serially infected (1∶5, 1∶25, 1∶125, and 1∶625) with ODVs released from OBs. ODV titers (ODV/ml) were calculated by end point dilution. Error bars indicate the standard error of the mean. d) Kaplan-Meier survival curves showing estimates of that the probability of an infected *S. frugiperda* larva surviving to different intervals following infection by each virus. Continuous and discontinuous lines represent Sfbac and Sf32null survival curves, respectively. e) OB production values in larvae infected with Sfbac and Sf32null viruses. Values above the columns indicate means. Error bars indicate the standard error of the mean. f) Dynamics of BV production through the time. Squares represent Sfbac values and triangles represent Sf32null values. Error bars indicate the standard deviation. No significant differences were observed in BV temporal production patterns between Sfbac and Sf32null viruses (p>0.05).

Intrahemocelic injection of Sf32null bacmid DNA resulted in significantly lower mortality of larvae than observed following injection of Sfbac or Sf32null-repair DNA (F_2,8_ = 25.878, p = 0.001). Mean (±SE) mortality was 15.7±1.3% for insects injected with Sf32null bacmid DNA, whereas 49.1±4.5% and 53.8±5.3% was recorded for those injected with Sfbac and Sf32null-repair DNA respectively (Figure 2a), indicating that Sf32null DNA was approximately three fold less infectious than viral DNA that included the gene. DNA extracted from the resulting OBs was subjected to restriction endonuclease analysis and PCR which confirmed that the three viruses had similar DNA restriction profiles and amplification products to those obtained with the parental DNA.

### Sf32null Bacmid OBs have an Increased DNA Content and Similar ODV Content

DNA content was quantified by qPCR (efficiency = 101%, r^2^ = 0.998). The presence of non-specific amplification resulting in the high PCR efficiency was considered unimportant, as only one peak could be observed in the melting curve. Significant differences were observed in the mean concentration of DNA in OB samples from Sfbac, Sf32null and Sf32null-repair (F_2,30_ = 7.468, p = 0.002). Sf32null OBs yielded an average (±SE) of 148.4±18.0 ng DNA/10^6^ OBs, that was significantly more DNA than Sfbac (91.5±13.6 ng DNA/10^6^ OBs) or Sf32null-repair OBs (72.7±9.0 ng DNA/10^6^ OBs) (Figure 2b). However, similar titers of infectious units (ODVs) were present in Sfbac and Sf32null viruses in standardized samples of 5×10^8^ OBs (t = 0.28, df = 10, p>0.05). In this case, the ODV titers were 6.6×10^3^±1.5×10^3^ infectious units/ml for Sfbac compared with 7.4×10^3^±2.6×10^3^ infectious units/ml for the Sf32null virus (Figure 2c). The complete recovery of biological activity in the Sf32null-repair virus in terms of DNA infectivity and DNA content led us to use only the Sfbac as a control virus in the following experiments.

### Deletion of *sf32* had no Significant Effects on OB Pathogenicity or Speed of Kill but Increased OB Production

The estimated LC_50_ values of Sfbac and Sf32null bacmid OBs were almost identical at 1.76×10^4^ and 1.77×10^4^ OBs/mL, respectively. The 95% confidence levels of the relative potencies, representing the ratio of effective concentrations [36], overlapped broadly in both viruses indicating no significant differences in OB infectivity between these viruses in *S. frugiperda* larvae (Table 2).

**Table 2:** LC_50_ values and mean time to death (MTD) for Sfbac and Sf32null in second instar *S. frugiperda* larvae.

Virus	LC_50_	Relative	95% Confidence limits		MTD	95% Confidence limits	
	(OBs/ml)	Potency	Low	High	(h)	Low	High
**Sfbac**	1.76×10^4^	1.00	−	−	161a	157	165
**Sf32null**	1.77×10^4^	0.99	0.66	1.50	161a	157	166

Probit analysis was performed using the PoloPlus program. The hypothesis of equality was not rejected (χ^2^ = 0.01, df = 2, p = 0.997) and a test for nonparallelism was not significant (χ^2^ = 0.01, df = 1, p = 0.947) such that regressions were fitted with a common slope of 1.163±0.092 (mean ± S.E.). Relative potency was calculated as the ratio of effective concentrations relative to Sfbac OBs. Mean time to death values (MTDs) were estimated by Weibull analysis [29]. MTDs labeled with same letter did not differ significantly (p>0.05).

Following inoculation with an estimated LC_90_ concentration of OBs the mean (±SE) observed mortality of second instars was 86.7±4.8% for Sfbac and 86.5±3.6% for Sf32null. The mean time to death (MTD) value was 161 hours post infection (hpi), which was identical for both viruses (Table 2). Mortality results were also subjected to survival analysis in SPSS 15.0. Kaplan-Meier curves (Figure 2d) and Log Rank test (χ^2^ = 0.210, df = 1, p = 0.647), which confirmed that the speed-of-kill was similar in both viruses.

OB production differed significantly between Sfbac and Sf32null viruses (t = 6.6; df = 4; p = 0.003). Sfbac infections produced 8.68×10^7^±0.57×10^7^ OB/larva (mean ± SE) whereas Sf32null produced 5.29×10^7^±0.42×10^7^ OB/larva (Figure 2e), which represents 39% fewer Sf32null OBs/larva compared to the production observed in insects infected by Sfbac.

*Sf32* had no pronounced effects on budded virus production (Figure 2f). The amounts of viral genomic DNA in hemolymph for both viruses at different times post-infection were very similar as determined by qPCR (efficiency = 108%, r^2^ = 0.998), i.e., *sf32* deletion appeared to have no clear influence on BV production at any stage of the infection. The presence of non-specific amplification was considered improbable as only one peak was observed in the melting curve.

### Sf32null Virus OBs are Larger than those of Sfbac Virus and Occlude ODVs Containing a Higher Number of Nucleocapsids

**Figure 3:**
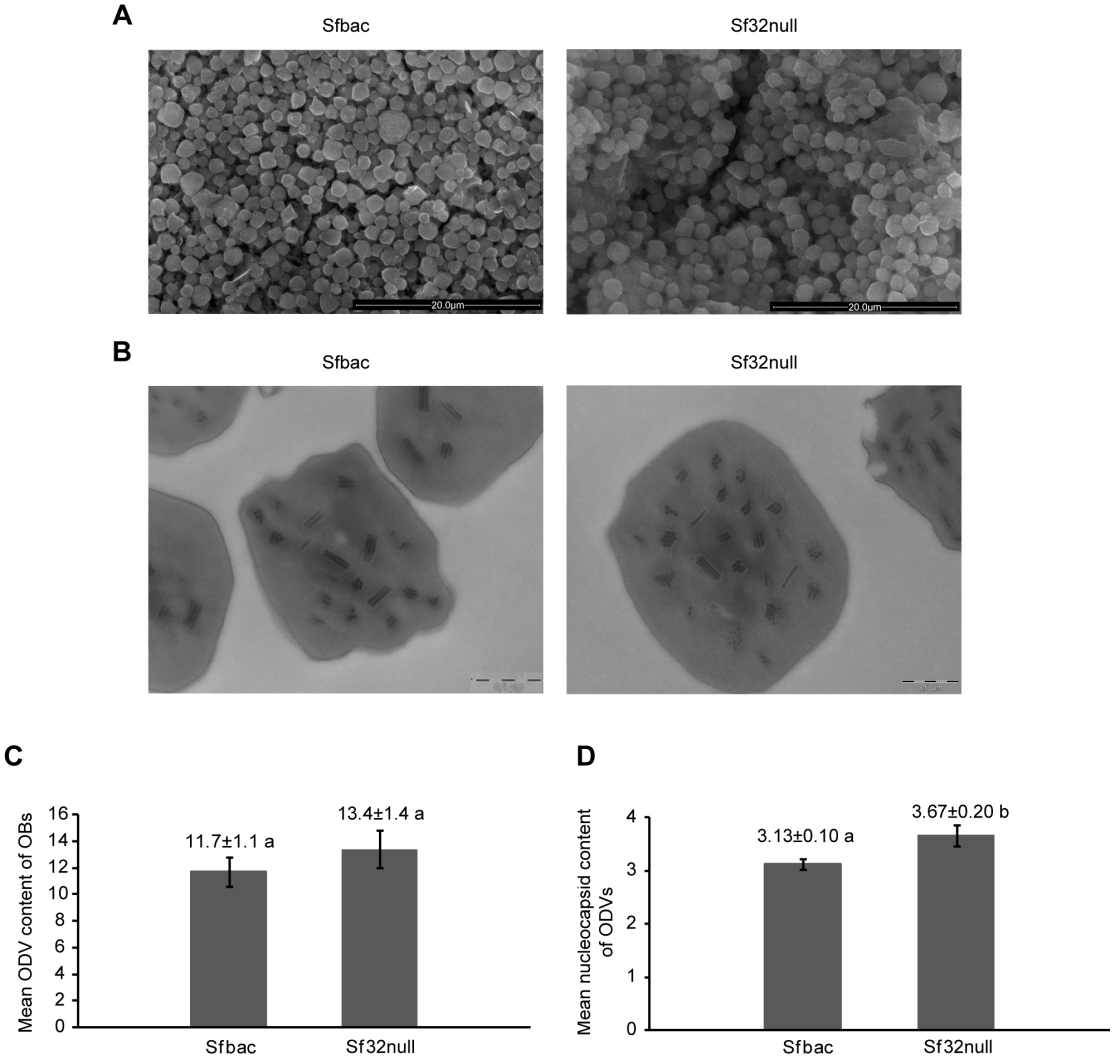
Electron microscopy of Sfbac and Sf32null OBs. A) Scanning electron microscopy (×6,000) of Sfbac and Sf32null OBs. *Sf32* deletion did not affect gross OB morphology although Sf32null OBs were approximately 18% larger in diameter than those of the Sfbac virus. B) Transmission electron microscopy (×40,000) of Sfbac and Sf32null OBs showing the distribution of single and multiple nucleocapsid ODVs. C) Mean number of ODVs occluded within OBs as determined by TEM analysis. D) Mean nucleocapsid content of ODVs estimated by analysis of OB sections following TEM. Values above columns indicate means and those labeled with different letters differed significantly (t-test, p<0.05). Error bars indicate the standard error of the mean.

Sf32null OBs had diameters between 1.40 and 3.37 µm with an average (± SE) value of 2.25±0.02 µm (N = 345), which was significantly larger than the diameter of Sfbac OBs, which ranged from 1.00 to 3.16 µm with an average value of 1.91±0.02 µm (N = 500) (t = 12.3, df = 843, p<0.001). Although Sf32null OBs were approximately 18% larger than those of Sfbac, no additional gross morphological differences were observed in OB structure between these viruses when examined by SEM (Figure 3A).

TEM observation revealed that the number of ODVs present within the OBs was similar in Sfbac and Sf32null OBs (t = 1.367, df = 26, p = 0.183) (Figure 3B, C). However, the number of nucleocapsids per ODV was approximately 17% higher in Sf32null ODVs than in Sfbac ODVs (t = 2.513, df = 311, p = 0.013) (Figure 3D). Significant differences in the size of the OBs were confirmed by measuring the Feret’s diameter in the TEM images (t = 3.47, df = 184, p = 0.001). The mean diameter of the Sfbac OBs was 1.64±0.04 µm, whereas the mean diameter of the Sf32null OBs was 1.84±0.04 µm.

### Sequence Analysis

Sequence analysis revealed that *sf32* is a reverse gene located between nucleotides 30,957 and 31,475 in the SfMNPV Nicaraguan isolate genome [14]. This gene is positioned between *sf31*, which encodes a putative protein-kinase interacting protein similar to *ac24,* and *sf33*, a putative actin rearrangement-inducing factor similar to *ac20* and *ac21*. A TATA box and a baculovirus consensus early promoter motif CAGT were present at 277 and 292 nt upstream from the initiation codon (ATG), respectively, suggesting that *sf32* might be an early gene, which was confirmed by RT-PCR temporal expression analysis (Figure 1).

SF32 is a unique small protein of 172 amino acids (Aa), present in all sequenced SfMNPV genotypes; no homologs were detected in other baculoviruses. The SF32 protein showed 26% identity and 44% similarity to the trypsin-resistant T6 surface protein of *Streptococcus pyogenes* serotype M6 and also showed sequence similarity with the fimbrial structural subunit of the same bacterial species (26% identity, 44% similarity). No signal peptide or transmembrane domains were detected in the putative protein.

**Figure 4:**
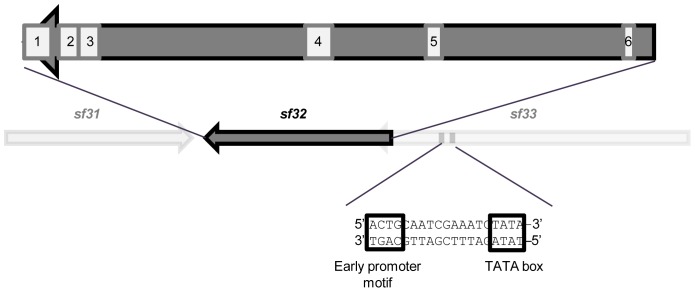
Sequence analysis of *sf32*. An early promoter motif and a TATA box were found upstream from the initiation codon. Numbers indicate predicted post-translational modifications and functional motifs of the protein. 1: N-myristoylation site; 2: casein-kinase II phosphorylation site; 3 and 5: N-glycosylation sites; 4: tyrosine-kinase phosphorylation site; 6: protein kinase C phosphorylation site.

The estimated volume of this protein was 24,688 A^3^ using the Peptide Property Calculator (Chazan). Secondary structure prediction revealed nine strands and five helices, but larger structures could not be predicted from these data. The PROSITE tool predicted some post-translational modifications and functional motifs of SF32, including a protein kinase C phosphorylation site (Aa7–9), two N-glycosylation sites (Aa60–63, 156–159), a tyrosine-kinase phosphorylation site (Aa89), a casein-kinase II phosphorylation site (Aa158–161) and an N-myristoylation site (Aa166–171) (Figure 4).

## Discussion

Baculoviruses may acquire genes from their hosts or from other coinfecting agents resulting in viruses with novel phenotypes [18]. Such modified phenotypic characteristics may confer a selective advantage related to the host range of the virus, its insecticidal activity or its transmissibility [17], [19], [37]. SfMNPV has twelve ORFs which have no homologs in other sequenced baculoviruses [13]. The number of unique genes varies between baculoviruses: 24 unique ORFs were identified in the genome of *Chrysodeixis chalcites* NPV (ChchNPV) [19], 20 unique ORFs were identified in the *Helicoverpa armigera* NPV (HaNPV) genome [38], whereas only three genes are unique to the AcMNPV genome [1], [39].

In order to increase our understanding of SfMNPV as a fall armyworm pathogen, the role of the *sf32* unique gene in the replication and transmission of this virus was studied by producing bacmid-based mutants that lacked the gene. Transcription of *sf32* starts very early during infection and continues for at least 144 hours. The temporal expression observations are in agreement with the presence of the early promoter detected by sequence analysis. The products of baculovirus early genes are often involved in DNA replication, regulation of late gene expression and host-modification processes [40]. Early gene transcription is mediated by the host RNA polymerase II and is strongly influenced by the immediate-early IE-1 and IE-2 factors [40]. Deletion of *sf32* resulted in a three-fold decrease in DNA infectivity of the bacmid virus, for reasons that remain unclear. Nonetheless, this early gene is clearly not essential for virus replication, as infectious OB progeny were obtained from the *sf32* deletion bacmid.

A 62% increase in the average amount of DNA per OB was observed in the Sf32null recombinant, but a similar number of ODVs were present in both Sfbac and Sf32null OBs. Other early genes, such as *ac23*, modify the number of nucleocapsids per OB without affecting the number of occluded ODVs [41]. In this case it can be assumed that gene deletion decreased total DNA content, in contrast to that observed with the *sf32* deletion mutant. The role of *sf32* in the SfMNPV virus therefore differs from that of *ac23* in AcMNPV.

Despite the increased DNA content of Sf32null OBs, *sf32* deletion had a negligible effect on OB pathogenicity or speed-of-kill. This was an unexpected result given that when a larva ingests an Sf32null OB it will be exposed to a greater number of viral genomes than occurs following ingestion of an Sfbac OB. However, deletion of the previously mentioned *ac23*, which also modifies the DNA content within OBs, had no significant influence on OB pathogenicity but increased the mean time to death of infected insects [42], so that changes in the DNA content of OBs are not neccessarilly linked to changes in their pathogenicity.

Interestingly, Sf32null OBs were 18% larger in diameter than those of the complete virus. This difference may not seem particularly important, but an 18% increase in diameter represents a 60% increase in volume of the OB (assuming that an OB approximates to the shape of a sphere), which is very similar to the observed increase in the DNA content of Sf32null OBs.

The total production of OBs/larva was ∼39% lower in Sf32null infected insects compared with those infected with the complete virus, whereas BV production was similar in both viruses. Increased OB productivity is often linked to an extended period of infection and an increase in the mean time to death [24], [43], [44], but this was not observed in insects infected by the Sf32null virus. Altered OB production could also be the consequence of alterations in the packaging and occlusion process. Clear similarities in the dynamics of BV production between viruses suggested that DNA synthesis was not affected by *sf32* deletion, yet Sf32null OBs contained approximately 62% more DNA than Sfbac OBs. This led us to suspect that more ODVs would be occluded in Sf32null OBs compared to Sfbac OBs, or a greater number of nucleocapsids would be enveloped in the Sf32null ODVs. The number of virions occluded within OBs was determined in cell culture and by direct TEM observation of OB sections, and was not affected significantly by deletion of *sf32*. However, deletion of this gene resulted in a significant increase in the number of viral nucleocapsids enveloped within each ODV, which reflects the higher DNA content of Sf32null OBs. Therefore, it appears that *sf32* likely affects nucleocapsid organization during ODV assembly and occlusion. The higher nucleocapsid content of Sf32null OBs compared to that of Sfbac may be responsible for the reduced OB production, as more genomic DNA is required for production of Sf32null OBs than in Sfbac OBs. Whether or not the increase in the number of nucleocapsids per ODV has a direct influence on the occlusion process, leading to the physically larger OBs that we observed, is unclear at present.

Analysis of the putative SF32 protein revealed that this protein does not contain any signal peptide or transmembrane domains, suggesting that is likely to be an intracellular polypeptide. Nevertheless, this protein is homologous to the trypsin-resistant T6 surface protein of *Streptococcus pyogenes* serotype M6 and the fimbrial structural subunit of the same species, both of which have adhesion functions [45]. The idea that SF32 could be involved in adhesion processes is an appealing concept that appears consistent with its hypothesized role in nucleocapsid organization during ODV assembly and occlusion. Some strains of *Streptococcus* are pathogenic to insects [46], [47] and horizontal transfer between viruses and bacteria has been proposed for other baculovirus genes, notably *chitinase* [48], [49]. The idea that SF32 may be involved in adhesion functions is reinforced by the presence of an N-myristoylation site in the amino acid sequence as this modification is usually related to weak and reversible protein-membrane and protein-protein interactions [50], [51]. In other viruses, myristoylated proteins are involved in assembly, structure, budding, intracellular host interactions and viral entry [52].

We conclude that the SfMNPV unique SF32 protein is a non-essential protein, as viral replication was not compromised by gene deletion. This gene might be directly or indirectly involved in mediating nucleocapsid organization during ODV assembly and occlusion. Deletion of *sf32* resulted in a reduction in OB production per insect and substantial increases in the size of OBs and an increase in the average number of nucleocapsids present within ODVs. Gene deletion did not affect OB pathogenicity, speed of kill, ODV content within OBs or the dynamics of BV production. Homology with bacterial adhesion proteins and the presence of an N-myristoylation site suggests that SF32 may affect nucleocapsid packaging in ODVs via interactions with other proteins.
